# Advanced 3Dimentional engineered microenvironment to improve of *in vitro* spermatogenesis

**DOI:** 10.5935/1518-0557.20240077

**Published:** 2025

**Authors:** Masoud Ghorbani, Roya Hassani, Mohammad Reza Nourani, Vahabodin Goodarzi

**Affiliations:** 1 Tissue Engineering and Regenerative Medicine Research Center, Baqiyatallah University of Medical Sciences, Tehran, Iran; 2 Department of Anatomy, School of Medicine, Iran University of Medical Sciences, Tehran, Iran

**Keywords:** 3dimentional, spermatogenesis, germ cells, stem cells

## Abstract

Induction of *in vitro* spermatogenesis may be helpful in the treatment of infertility in azoospermic individuals and those undergoing chemotherapy. Different cultivation systems have been implemented to achieve this aim. This review study aimed to investigate the application of three-dimensional culture in the induction of *in vitro* spermatogenesis. Relevant studies published in English were identified using PubMed using a range of search terms related to the core focus on tissue engineering of male reproductive systems, *in vitro* spermatogenesis, germ cell preservation, 3D culture systems for *in vitro* spermatogenesis, a 3D culture of testis tissue with were last updated in end of 2023. Searches were not restricted to a particular time frame or species, although the emphasis within the review is on regenerative medicine in mammalian male fertility preservation and *in vitro* spermatogenesis. Spermatogenesis is one of the most complicated cellular differentiation processes in the body. Significant attempts have been made to control spermatogenesis to drive differentiation of male germ stem cells toward mature sperm. Current research efforts focus on providing appropriate microenvironmental conditions to support the process of *in vitro* spermatogenesis by applying the principles of cell transplantation, material science, and bioengineering. Regenerative medicine may open a new avenue to patients for restoration and maintenance of normal function in spermatogenesis.

The techniques reviewed are still in development, and this paper can become the primary reference for a large body of scientists developing advanced tissue engineering for male germ cells or developing the next generation of reproductive medicine.

## INTRODUCTION

**Figure 1:**
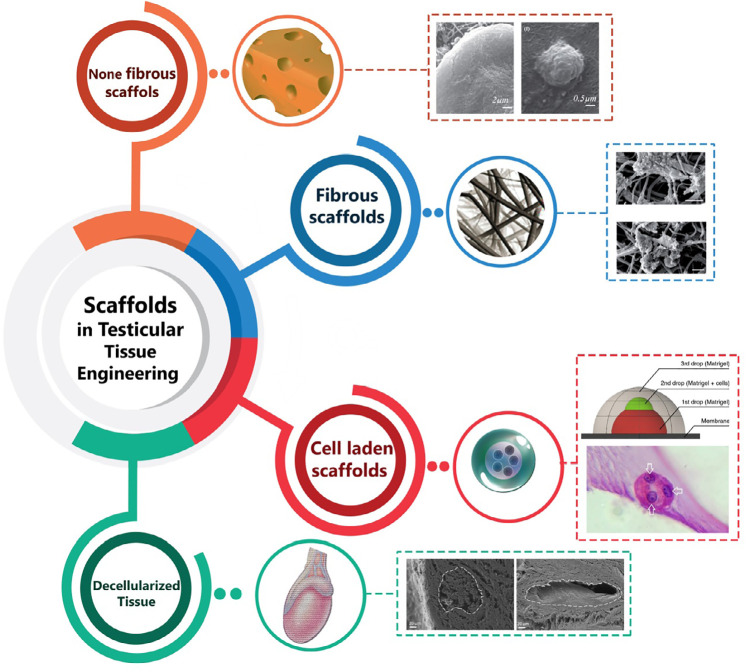
Application of advanced technologies in male fertility preservation and *in vitro* spermatogenesis, utilizing 3D engineered microenvironment.

Spermatogenesis is a complex process of proliferation and differentiation of germ cells that leads to the production of fertile sperm and includes different types of undifferentiated and differentiated cells located inside the seminiferous tubules of the testis. Spermatogonia stem cells are diploid cells that attach to the basement membrane of seminiferous tubules, initiating the process of spermatogenesis and maintaining it throughout adulthood (Houda *et al*., 2021). These cells divide inside the seminiferous tubules of the testis and form two types of daughter cells, including new stem cells and progenitor cells. This process is controlled *in vivo* by various factors, including hormones, growth factors, cytokines, and extracellular matrix proteins. Each of these factors interacts between Sertoli cells, and germ cells can affect the spermatogenesis process. Deficiency of any of these factors can lead to male infertility (Khanehzad *et al*., 2021). *In vivo* and *in vitro* transplantation of stem cells can be used to treat male infertility. *In vivo* transplantation is not possible in cancer patients undergoing chemotherapy due to the risk of cancer cells returning to the body. Many efforts are being made to *in vitro* differentiate spermatogonia from adult sperm. *In vitro* differentiation of spermatogonia stem cells and production of fertile sperm requires a suitable culture medium and microenvironment to support cell proliferation and differentiation. Among rudiments that compose the tissue microenvironment as growth factors, hormones, and other biological element, the extracellular matrix (ECM) stands out for its role in testicular tissue homeostasis (Porzionato *et al*., 2018; Ashammakhi *et al*., 2022; Ibtisham & Honaramooz, 2020). The ECM also provides three dimensions that enhance the interaction between the cell and the extracellular environment and increase the cell’s sensitivity to molecular signals from the ECM and other exogenous agents (Porzionato *et al*., 2018). Therefore, the successful *in vitro* spermatogenesis requires suitable 3D microenvironment and 3D engineered scaffolds can play an effective role in simulating the extracellular matrix and supporting the proliferation and differentiation of germ cells (Ashammakhi *et al*., 2022; Cortez *et al*., 2022). The technologies described in this review will be the key to unborn results making use of 3D culture and advanced pulpits to give *in vitro* spermatogenesis and manly fertility preservation. These technologies can give better mimics of the species-specific and age-specific arrangements of the testis and biomechanical and biochemical parcels of the mammalian reproductive tract and may overcome the defects of *in vitro* 2D culture vessels (Figure 1).

## SPERMATOGENESIS ON IN VIVO AND *IN VITRO* MICROENVIRONMENT

Although research into the process of spermatogenesis in the laboratory has been underway since the early part of the last century, the differentiation of spermatogonia stem cells into sperm remains a challenge. The process of spermatogenesis occurs *in vivo* inside the seminiferous tubules of the testis. During this process, the spermatogonia stem cells divide and form two types of daughter cells, including new stem cells and progenitor cells. This trend varies in different species of mammals. In all species, a small population of testicular stem cells acts as a reservoir with a high capacity for colonization of seminiferous tubules (Ungewitter & Yao, 2013; Fayomi & Orwig, 2018). Stem cells are active in each cycle of the seminiferous tubular epithelium (Fayomi & Orwig, 2018). Paracrine and autocrine hormones and factors control the process of spermatogenesis (Cham *et al*., 2021). One of the essential factors, in this case, is the interaction between the developing germ cells and the surrounding supporting cells such as Sertoli. Peritubular cells and various interstitial cells, such as Leydig cells, macrophages, and endothelial cells, regulate the formation of germ cells by secreting various factors (Guo *et al*., 2020; Di Persio & Neuhaus, 2023), which should be considered in the induction of *in vitro* spermatogenesis. Over the years, various methods have been proposed to induce the process of spermatogenesis, including two-dimensional culture, three-dimensional culture, testicular tissue culture on agarose gel, and cell transplantation to azoospermia testis and cell culture using testicular scaffolds.

## CULTURE OF SEMINIFEROUS TUBULES

*In vitro* organ culture systems are considered as applicable models for the disquisition of pathophysiological mechanisms which can directly mimic the functions of an organ in colorful countries and conditions (de Rooij, 2017). By cultivating towel fractions or entire organ *in vitro*, the towel structure can be saved to support the natural experimental processes (Mohaqiq *et al*., 2019a). Organ societies give an occasion to manipulate the paracrine terrain and also to examine the part of each growth factor collectively on the spermatogenesis process (Song & Wilkinson, 2012).

The 3D testicular tissue culture systems are applicable for spermatogenesis progress as they can maintain the commerce of the seminiferous tubules and interstitial area (Shams *et al*., 2017). It seems that this system can be used to induce and renew spermatogenesis by *in vitro* SSC transplantation, in order to produce mature sperm for highposition remedial reproductive medicine operations (Galdon *et al*., 2016). Although the applicable conditions for culture of testis tissue and testicular cells are different, the media used for organ culture are generally the same as those used for cell growth. still, analogous media need to be optimized by adding specific essential and effective ingredients (analogous as retinoic acid, luteinizing hormone, FSH, testosterone, or other feathers of vitamins, antioxidants, hormones, and growth factors) to promote *in vitro* spermatogenesis (Sanjo *et al*., 2018).

**Figure 2:**
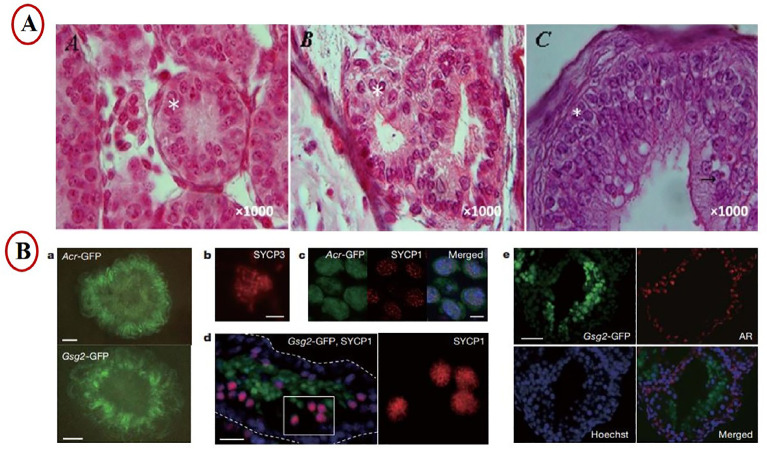
Experimental methods schematic presentation of in vitro spermatogenesis using culture of seminiferous tubules. A) Histological section of the testis tissue fragment cultured for (A) 8 weeks, (B) 10 weeks, and (C) 12 weeks. Stars and the arrow demonstrate spermatogonial cell and spermatocyte cell during culture, respectively (Alrahel *et al*., 2018). B) a, 10% KSR induced the expression of both Acr-GFP and Gsg2-GFP in 2.5 and 0.5 dpp mouse testes, respectively. b, Immunostaining with anti-SYCP3 antibody. c, AcrGFP-expressing cells (green) at the pachytene stage were also stained with SYCP1 (red). d, In a cryosection of a Gsg2-GFP testis tissue, SYCP1 was demonstrated in cells (red) outside the Gsg2-GFPpositive cells (green). Hoechst (blue). e, Gsg2-GFP-expressing testis tissue, originating from 2.5 dpp mice and cultured for 21 days, was cryosectioned and stained with antibodies against GFP, AR, and counterstained with Hoechst dye (Sato *et al*., 2011).

In the past, the study of spermatogenesis *in vitro* began with the culturing of testicular tissue from a rodent infant. The main advantage of this approach is that the germ cells maintain their cellular arrangement during growth (Sanjo *et al*., 2018; Sakib *et al*., 2019; 2020). To a large extent, these studies failed to demonstrate spermatogenesis beyond the mitotic stages. However, Sato *et al*. (2011) developed a protocol that showed spermatogenesis in the laboratory and improved the differentiation of stem cells by spermatogonia and sperm formation using testicular tissue culture. They were differentiated into fertile sperm. Alrahel *et al*. (2018) were also able to prove the expression of post-meiotic genes such as Tnp1 at the molecular level by culturing mouse testis fragments on agarose gel and using various growth factors (Figure 2).

## SPERMATOGONIAL STEM CELL TRANSPLANTATION

Spermatogonial stem cell transplantation was first performed by Brinster and Zimmermann to confirm the presence and function of spermatogonial stem cells. Injection of these cells into the testes of busulfantreated mice showed that the cells migrated to the seminiferous tubules and participated in the complete spermatogenesis to produce rich sperm. The product of mature sperm by transplanted stem cells indicates the significance of a specific testicular medium in the process of spermatogenesis (Brinster & Zimmermann, 1994). Absalan *et al*. (2011) transplanted spermatogonial stem cells into the testes of cryptorchidism mice. Their study showed that in transplanted groups, sperm production increased significantly compared to the non-transplanted group. Koruji *et al*. (2012) transplanted fresh and frozen-thawed mouse spermatogonia stem cells into autogenous gamma testis. Their study showed that the mean percentage of filling of seminiferous tubules in transplanted groups was significantly different from non-transplanted groups. Spermatogonia stem cells also produced sperm after transplantation.

**Figure 3:**
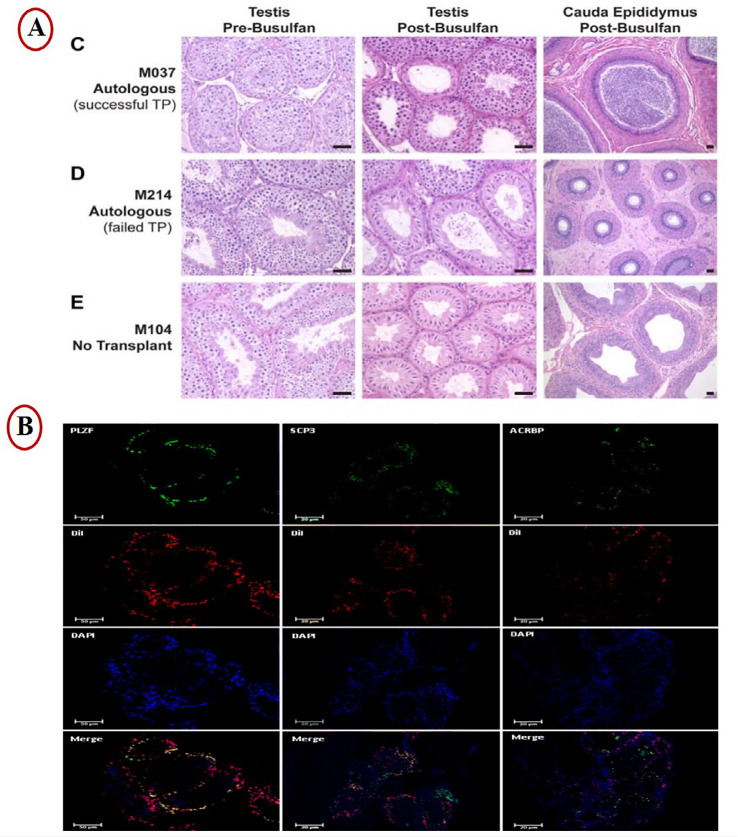
Potential clinical applications of the spermatogonial stem cell transplantation. A) (C) M037, which exhibited successful transplant engraftment based on presence of sperm in the ejaculate, (D) transplant recipient M214 which never exhibited sperm in the ejaculate after transplant, (E) Histology from the testis and epididymis of an un-transplanted animal M104 illustrates the appearance of an azoospermic (empty) testis after busulfan treatment (Hermann *et al*., 2012). B) Immunohistochemistry of host testes after transplantation and organ culture. Expression of specifc proteins of spermatogonial cells (PLZF), spermatocytes (SCP3) and spermatozoa (ACRBP) and detection of DiI in host testes after 8 weeks of tissue culture (Mohaqiq *et al*., 2019a).

Another group of researchers performed the technique of transplanting spermatogonia into rhesus monkeys. They reported that spermatogenic stem cells injected into seminiferous tubules differentiated into sperm (Hermann *et al*., 2012). Mohaqiq *et al*. (2019b) isolated spermatogonia stem cells from testicular tissues and injected azoospermic testis into the seminiferous tubules. The results of their study showed that after two weeks, spermatogonial stem cells settled on the basement membrane of seminiferous tubules (Mohaqiq *et al*., 2019b). The group also injected spermatogonia stem cells isolated from frozen-thawed testicular tissue into azoospermic testes, cultured in agarose gel *in vitro* for eight weeks, and reported haploid cell production (Mohaqiq *et al*., 2019a) (Figure 3).

## 3D CULTURE TO INDUCTION OF SPERMATOGENESIS

3D culture systems were first developed to evaluate the colonization of hematopoietic stem cells and to discover the complex mechanisms involved in their proliferation and differentiation (Zmrhal *et al*., 2022; Congrains *et al*., 2021). Adaption of this approach to the manly reproductive system handed irrefragable substantiation that testicular origin cells could separate outside the body to the stage of stretched spermatids (Ganjibakhsh *et al*., 2019; AbuMadighem *et al*., 2022). Threedimensional culture of testicular origin cells can mimic the medium of seminiferous tubules epithelium. This system increases our understanding of the commerce between Sertoli cells and origin cells and between the origin cells and the extracellular matrix, and the effect of these relations on the process of spermatogenesis. The essential part of Sertoli cells and extracellular matrix in survival, origin cell isolation is well known (Salem *et al*., 2023; Eyni *et al*., 2021).

3D culture *in vitro* can be accomplished by placing cells in scaffolding. Scaffolds facilitate cell adhesion, proliferation, and migration, the release of biochemical factors and nutrients. Artificial polymers such as polyglycolic acid, natural biomaterials such as collagen and alginate, and a cell-free natural matrix can be used to make scaffolds (Ghorbani *et al*., 2019; Campuzano & Pelling, 2019). Natural biomaterials for scaffold development can include extracellular matrix components such as collagen, fibrinogen, and hyaluronic acid. These scaffolds have advantages such as biocompatibility, bioactivity, and mechanical properties similar to natural texture. Other natural biomaterials such as cellulose, chitosan, and silk fibrin are obtained from plants and insects. Today, natural polymers are used in the form of viscous gel suspensions or the form of porous sponges (Saydé *et al*., 2021; Park *et al*., 2021; Johnston *et al*., 2023).

Collagen is one of the most abundant proteins found in the body. This polymer is a fibrous protein and a significant component of the extracellular matrix. For this reason, collagen is used in tissue regeneration, especially soft tissue. Common sources of collagen include cow or pigskin. Collagen contains side groups attached to the cell that these interactions may help maintain the appearance and activity of many cell types (Wang *et al*., 2023). Induction of the spermatogenesis process using natural biomaterial scaffolds is achieved by placing different cells isolated from seminiferous tubules in a collagen gel matrix. This provides adequate support for isolated spermatogonial to interact with Sertoli cells and other structural and hormone-producing elements. This method increases the survival of germ cells, the meiotic division of cells, and their differentiation into spermatid-like cells (Ashouri Movassagh *et al*., 2019; Kulibin & Malolina, 2023). Spermatogenesis has been reported in three-dimensional cultures in different species. For example, Lee *et al*. (2001; 2006; 2007) used scaffolds made of collagen and sodium alginate to induce spermatogenesis in isolated stem cells and succeeded in producing spermatids.

**Figure 4:**
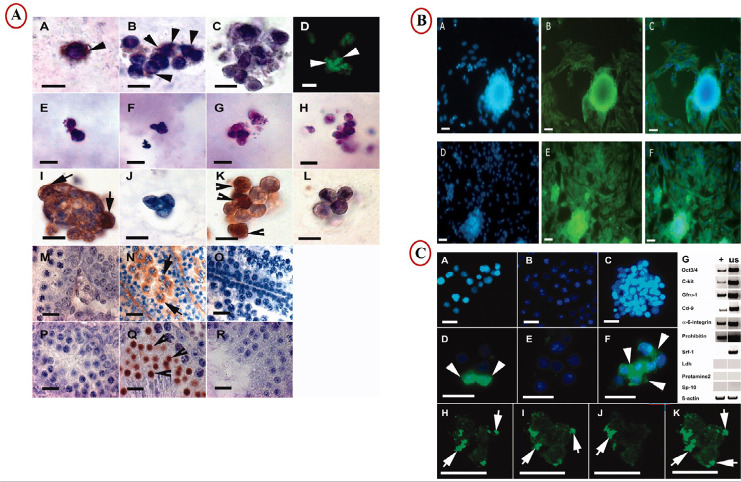
Advanced procedures of 3D culture to induction of spermatogenesis. A) Expression Gfrα-1 (A, B, D) in SACS-cultured cells. An image of a control labeling omitting a primary antibody is shown in C. Expression of the Boule protein (E-I) and Crem (K) in SACS-cultured cells and in the murine testis at different developmental stages (M-R) (Stukenborg *et al*., 2008). B) Representative immunofluorescence images (A) staining nucleus with DAPI (blue) and (B)showing SCP3 positive cells (green) (C) merge in control group (D, E, F) in co-culture group (Khajavi *et al*., 2014). C) A heterogeneous cell suspension containing single cells is observed in the unsorted cell fraction after digestion of cells before the separation procedure (A) and in the depleted fraction after MAC sorting with Gfrα-1 (B). A homogeneous cell suspension is observed in the enriched fraction (C). The mentioned fractions are shown after immunofluorescent labeling with anti-Gfrα-1 (unsorted fraction [D], depleted fraction [E], and enriched fraction [F]; arrowheads: Gfrα-1-positive cells [FITC]). Expression analysis of different spermatogenic marker genes (murine spermatogonial stages: Oct3/4, C-kit, Gfrα-1, Cd-9, and α-6-integrin; murine meiotic stages: Prohibitin and Srf-1; murine postmeiotic stages: Ldh, Protamine-2, and Sp-10; positive control: β-actin before (G; unsorted [us] lane) and after sorting with anti-GFRα-1 (G; enriched [+] lane). Confocal microscopy images of fluorescent-labeled cells showed expression of Gfrα-1 (arrows) on the cell surface of spermatogonial cells (H-J; overlay K) (Stukenborg *et al*., 2008).

Stukenborg *et al*. (2008) used a soft agar culture system (SACS) to culture spermatogonia. The soft agar culture system creates a seminiferous tubules epithelium-like microenvironment by providing a thick layer for spermatogonia stem cells and testicular somatic cells, inhibiting ischemia in long-term tissue growth. Khajavi *et al*. (2014) designed a three-dimensional culture system based on collagen gel extracted from rat tails. Mice spermatogonia were isolated by two steps of enzymatic digestion and MACS and divided into two groups. Group One: Spermatogonia cells were cultured in a three-dimensional culture medium of collagen gel without somatic cells. Group 2: Spermatogonia cells were cultured with Sertoli somatic cells and cells around the luminal. Their study showed that the co-culture of spermatogonia with somatic cells had positive effects on the expression of meiotic markers, post-meiosis, and colony formation (Khajavi *et al*., 2014) (Figure 4).

## CONCLUSION

Three-dimensional culture using scaffolds and stem cells can induce *in vitro* spermatogenesis. Also, decellularized and biocompatible testicular scaffolds have the potential to be used as a tool to study the process of spermatogenesis. However, there are technical and ethical challenges in the production of fertile sperm to treat disbelief that need further research.
